# Population correlations do not support the existence of set points for blood levels of calcium or glucose – a new model for homeostasis

**DOI:** 10.14814/phy2.13551

**Published:** 2018-01-12

**Authors:** Stephen P. Fitzgerald, Nigel G. Bean

**Affiliations:** ^1^ Royal Adelaide Hospital Adelaide South Australia Australia; ^2^ School of Mathematical Sciences and ARC Centre of Excellence for Mathematical and Statistical Frontiers University of Adelaide Adelaide South Australia Australia

**Keywords:** Calcium, glucose, homeostasis, set points

## Abstract

The prevailing teaching regarding homeostasis, and in particular endocrine homeostasis, includes the fundamental concept of a “set point,” which represents a target or optimum level defended by physiological control mechanisms. Analogies for the description and teaching of this concept have included thermostats and cruise controls. We previously demonstrated that such a set‐point model of regulation implies that in population data of parameter set point/controlling hormone levels, correlations between the parameter and its controlling hormone must be in the direction of the response of the parameter to its controlling hormone, and that in thyroid homeostasis this relationship is not observed. In this work we similarly examined population correlations, extracted from the literature, for the parameters glucose and calcium, and their controlling hormones. We found 10 correlations. Most were highly significant (*P* < 0.01). All were in the direction of the response of the controlling hormone to the parameter. Therefore, none were consistent with the pattern implied by a set‐point model of regulation. Instead all were consistent with an “equilibrium point” model of regulation, whereby ambient levels have no particular connotation to the individual, and result passively from the interplay of physiological processes. We conclude that glucose and calcium regulation, like thyroid regulation, are not centered on set points. This may reflect a general property of homeostasis. We provide an alternative mechanistic analogy, without a set point, for the heuristic description and teaching, of homeostasis.

## Introduction

“Homeostasis” is believed to be one of eight core concepts in biology and one of the two “big ideas” (concepts) in physiology (Modell et al. 2015). The current framework of the understanding of homeostasis is based on Claude Bernard's concept of the constancy of the “milieu interieur.” Walter Cannon subsequently introduced the term “homeostasis” and later still homeostatic regulatory mechanisms began to be described in terms of engineering and control system analysis (Modell et al. 2015; Goldstein, 2009). Thermostats and cruise controls in particular, have been used as analogies for homeostasis (Modell et al. 2015). Such an extrapolation of the mechanisms of familiar, everyday objects to physiological processes is a natural process of human cognition (Sloman and Fernbach 2017).

Homeostatic mechanisms maintain the stability of many physiological parameters about a certain value known as the “set point.” There is interindividual variation in the value of such set points, all of which are similarly defended (Romanovsky 2004; Curtis et al. 2007; Walsh 2011; Sherwood et al. 2013; Fliers et al. 2014; Leow and Goede 2014; Patton and Thibodeau 2016).

The term “set point” reflects the prevailing opinion that the stable levels of physiological parameters are akin to thermostat settings (Curtis et al. 2007; Walsh 2011; Hadlow et al. 2013; Sherwood et al. 2013; Fliers et al. 2014; Patton and Thibodeau 2016), or perhaps more accurately to an automobile's cruise control system (Modell et al. 2015), and thus set points have analogously been defined as “goal levels” (Goldstein 2009) or “the desired level at which homeostatic control mechanisms maintain a controlled variable” (Leow and Goede 2014). By extension the normal interindividual variation in set point values is explained by variation in this “desired level.”

All set‐point models require an obvious set point (a physical/physiological reference signal) or a hidden set point (a “mathematical” reference signal based on some internal property of the system) (Romanovsky 2004). Aside from the means to specify such a set point, such physiology also requires that the body has the means to identify the optimum level of a parameter to be so specified, a means to sense an “error signal” (a deviation from this set point), and a linked “corrective” process (Ashby 1957; Modell et al. 2015).

Set‐point models imply that, in the normal range, levels of a parameter are independent of the organ/processes producing the parameter, these organs in a sense being “slaves” (Leow and Goede 2014) to the set point process. Thus, the level of FT4 in the body is controlled and set by the hypothalamus‐pituitary rather than by the thyroid gland, and the level of calcium is controlled and set by the parathyroid glands rather than by the effector responses in the bone, kidney, and gut. The thyroid gland and the effector responses in the bone, kidney and gut in these two systems, act as directed by the hypothalamus‐pituitary and the parathyroid glands, respectively. Cannon proposed that the **brain** co‐ordinates body systems, with the aim of maintaining the goal values of key internal variables (Goldstein, 2009).

If homeostasis were likened to the movement of the earth around the sun, in the current model of homeostasis the set point is analogous to the sun that is at the center.

There are however recognized problems with this traditional model. These include that set points can be changeable, that in most cases the underlying molecular or cellular mechanisms cannot be identified, and that in different circumstances a physiological variable may be regulated or behave as a controlled variable (e.g., pCO2 in the different circumstances of respiration and acid‐base disturbance) (Modell et al. 2015). To counter these difficulties the model of homeostasis has become complex including such concepts of allostasis and hierarchies of control (Modell et al. 2015). The teaching of the principles of homeostasis has thus become challenging (Modell et al. 2015).

An alternative, not generally accepted, view regarding parameter regulation, is that parameter levels, rather than resulting from a set point, result simply from the balance of various homeostatic mechanisms at play (Partridge 1982; Romanovsky 2004). In this “equilibrium‐point model,” the level of the parameter has no particular meaning or connotation to the individual, and the interindividual variation in levels of a parameter results directly from interindividual variation in these homeostatic processes. (A set point too, results from a balance, but has the additional property that the balance is controlled so as to attain the target value. It is this additional property, an integral part of current teaching (Modell et al. 2015), which is the focus of this work).

The problems associated with our natural tendency to extrapolate mechanisms of action are well described (Sloman and Fernbach 2017), and in particular the cognitive error resulting in the extrapolation of engineering concepts to physiological processes shaped by evolution, has been exposed (Partridge 1982). Thus far however, this work and the experimental data supporting an equilibrium‐point model (Romanovsky 2004) have not diminished the dominance of the set‐point model.

In our previous work (Fitzgerald and Bean 2016), we mathematically analyzed thyroid homeostasis to clarify the difference between the curve describing (the line of best fit of) the population distribution of Free thyroxine/Thyroid Stimulating Hormone (FT4/TSH) levels (the “population curve”) and the curve describing the physiological suppression of TSH by FT4 in individuals.

**Figure 1:**
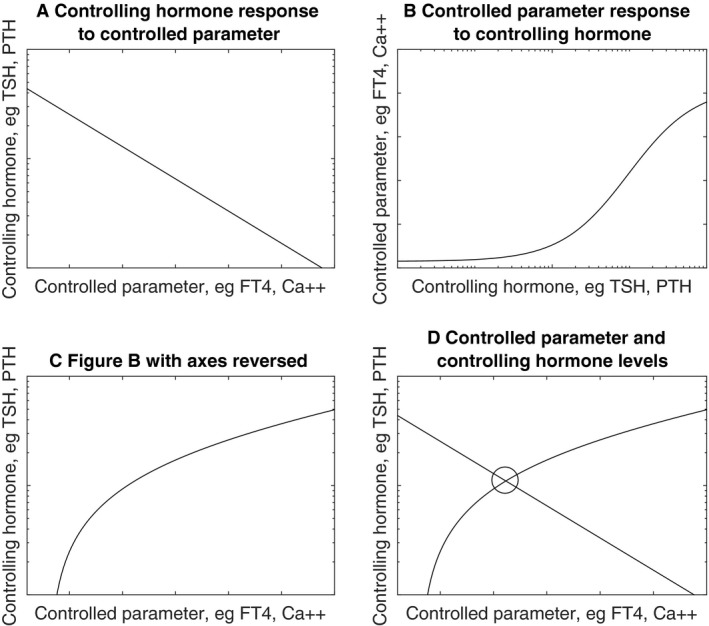
The negative feedback loop between a regulated parameter (e.g., FT4, calcium) and the respective controlling hormone (e.g., TSH, PTH). Panel {d} demonstrates that at equilibrium levels the two components of the feedback loop are solved simultaneously, as represented by the intersection point of the component curves. Different systems will differ in terms of the exact shapes of the component curves.

This work in turn was based on previous work showing that the ambient level of a parameter at steady state is such that it is at the intersection of the curves describing the components of the relevant feedback loops. Thus, in thyroid physiology FT4 is stimulated by TSH and TSH is inhibited by FT4. The ambient level of FT4 lies where the curves describing these two processes cross (Dietrich et al. 2012). For serum calcium levels the analogous point would be where the curves describing, the suppression of PTH by calcium, and the stimulation of calcium levels by PTH, cross (Fig. 1).

**Figure 2:**
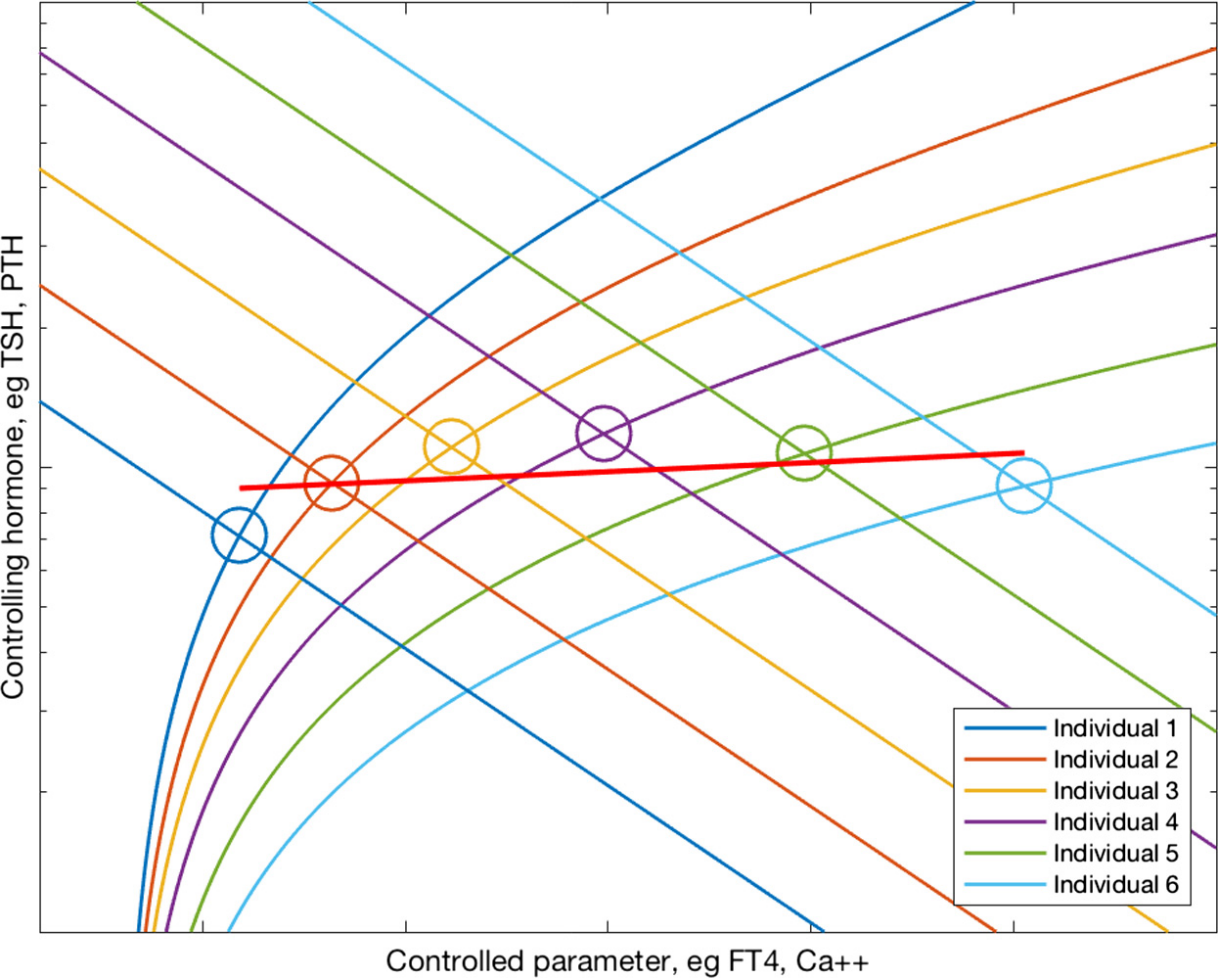
The extension of Figure 1{d}, demonstrating different individuals of a population attaining different levels of the same parameter, on account of interindividual variations in the components of the feedback loop leading to different intersection points. The red line indicates the derivation of a line of best fit, a “population curve.”

A population curve results from plotting all the different individual intersection points of all the individuals in a population, the different intersection points arising from interindividual variations in pituitary and thyroid (or the corresponding organs in calcium metabolism) responsiveness (Fig. 2) (Fitzgerald and Bean 2016). The pattern of these variations determines the shape of the population curve (Fitzgerald and Bean 2016).

Furthermore, we showed (Fitzgerald et al. 2017) that in a set‐point model of regulation the population curve must have a slope similar in direction to the curve describing the effect of the controlling hormone on the controlled parameter. In the case of the FT4/TSH relationship, the population curve in a set‐point model should therefore have a positive slope, reflecting the stimulatory effect of TSH on FT4 levels.

The principles underlying this conclusion are that if FT4 set point levels are set by adjustment of TSH by the hypothalamus‐pituitary (Goede et al. 2014; Leow and Goede 2014), they cannot simultaneously be set by non‐TSH related mechanisms. They must therefore be independent of interindividual variations (not due to TSH), in thyroid gland sensitivity to TSH. In these circumstances, individuals with higher set points must modulate their TSH response to FT4, so that, on average, they generate higher TSH levels, so as to achieve these higher FT4 set points.

Heuristically this can be understood by imagining a situation in which all members of the population have FT4 levels that have not been set, and result merely from the actions of feedback loops (equilibrium‐point model). We can then add to this situation the proposition that different individuals might function better with different specific levels of FT4 for any number of reasons including different metabolic requirements, different tissue sensitivities to FT4, different FT4 to FT3 conversion systems etc. Thus, each individual might become endowed with a different set point of FT4 which the body strives to achieve and maintain. This set point need not be fixed; it can be adjusted as appropriate to the individual's changing circumstances (Cabanac 2006; Fliers et al. 2014).

In these circumstances given that the control of the FT4 level is via hypothalamic‐pituitary mechanisms, the only way each individual's FT4 set point level can be attained is by adjustment of the TSH level. Thus, the only way individuals with a high‐normal FT4 set point can attain and maintain this set point is by running a high‐normal TSH level and conversely those individuals with a relatively low FT4 set point can only attain and maintain this level by running a relatively low TSH level. It follows that there must be a positive correlation in the population between FT4 and TSH.

Note that this relationship is the opposite of the more familiar relationship of higher FT4 values being associated with lower TSH values. This latter relationship reflects a compensatory process and occurs in an individual when the FT4 value is raised from a given level as a result of changes to the thyroid gland or FT4 metabolism (Dietrich et al. 2012; Leow and Goede 2014). This is a different phenomenon to a rise in the FT4 level being caused by a rise in the TSH level. Both mechanisms of raising FT4 exist in thyroid physiology and analogous processes occur in other endocrine systems (Foresta et al. 1997; Andersen et al. 2002; Fliers et al. 2014). It is in fact the manipulation of the TSH response to FT4 which is invoked to explain the phenomenon of an adjustment to the FT4 set point (Fliers et al. 2014). The fact that attainment of any initial set point must too involve such manipulation of TSH has been recognized (Goede et al. 2014; Leow and Goede 2014), but it seems that we were the first to recognize how such manipulation, if present, must affect population correlations (Fitzgerald et al. 2017).

We are even more familiar with the stimulatory relationship between a parameter and its regulating hormone in the situations of ACTH driving cortisol, and gonadotropins driving sex hormones (situations where there can indeed be a positive correlation between the variables (Bando et al. 1991; Foresta et al. 1997; Miro et al. 2004)).

Though the slope, in terms of being positive or negative, of a population curve is restricted in the set‐point model, any slope of the population curve is consistent with an equilibrium‐point model, as in this model the slope merely depends on the distribution across the population of the characteristics of the relevant feedback loop (Fitzgerald and Bean 2016).

As the empirically derived population curve for FT4/TSH has a negative slope (Hoermann et al. 2010; Hadlow et al. 2013) (on average, individuals with higher levels of FT4 have lower levels of TSH) it indicates that FT4 regulation is consistent with an equilibrium‐point model but not with a set‐point model.

In this work we extended our analysis to other systems with putative set points. We studied the regulation of glucose/insulin, and calcium/parathyroid hormone (PTH), so as to determine whether or not the nature of thyroid regulation is typical of regulation in endocrinology and homeostasis in general.

On account of our results, and in part so they might be more easily comprehended, we also subsequently developed an alternative analogy/model of a heating system, not based on a thermostat, which better describes the principles of homeostasis. This analogy could equally be applied to a cruise control system etc. This analogy further demonstrates how the model of homeostasis has implications for the population correlations between a parameter and its controlling hormone.

## Method

We sought data on the population distributions of glucose/insulin, and calcium/PTH. We restricted our analysis to the normal range. Our experience in studying thyroid homeostasis was that such data might be available from studies directed to various research questions unrelated to our enquiry. We conducted searches of the literature using the search engines PubMed, Embase, Scopus, and Google Scholar. We used search terms “calcium,” “parathyroid hormone,” “insulin,” “glucose,” “correlation,” and “population.” Of the resulting hundreds of articles, most were excluded on review of the abstract, on account of them not appearing to address our subject of interest. We examined more closely articles seemingly more related to our subject matter, and pursued references in these articles. We contacted authors when access to data and additional analyses appeared warranted.

In our previous work on thyroid regulation the empiric population data were expressed in terms of a curve of best fit. In terms of the population distribution of calcium/PTH and glucose/insulin we similarly sought curves of best fit. It became apparent that the data we sought were more often expressed as a correlation. We considered that, for our purposes, both approaches gave the same information. We were interested only in whether, in the normal range, the slope of the line of best fit, or the correlations, were positive, negative, or zero.

We then compared these slopes or correlations, with those that would be implied by a set‐point model of regulation. Though in our earlier work on the thyroid we showed that in a set‐point model of regulation population correlations must be in the direction of the response FT4 to TSH, the generalization of this principle is that the population correlations must be in the direction of the controlled parameter to the controlling hormone (Fitzgerald et al. 2016). Therefore, such a model implies that, as calcium levels are *stimulated* by PTH, the population correlations of calcium and PTH must be positive. In the case of glucose/insulin, the glucose level is *suppressed* by its regulatory hormone insulin and thus the correlations in a set‐point model should also be reversed; that is they should be negative. (Using the same heuristic as above if an individual has a relatively low set point for the blood glucose level, and if this level is dependent on the blood insulin level, this individual must secrete relatively high levels of insulin so as to attain the relatively low blood glucose set point.)

## Results

We found two studies concerning the population calcium/PTH relationship (*n* = 946,115) (Minisola et al. 1993; Jorde et al. 1999), and three studies concerning the population glucose/insulin relationship (*n* = 7074, 3247, 290) (Gibson et al. 1975; Chen et al. 2005; Peplies et al. 2014).

None of the studies we retrieved specifically addressed the set point/equilibrium point distinction. The data concerning the calcium/PTH relationship were collected to investigate a relationship between PTH and blood pressure (Jorde et al. 1999), and to clarify interrelationships between age and sex within the parathyroid endocrine system with a view to contributing to the study of age related bone loss (Minisola et al. 1993). All of the data concerning glucose/insulin were collected to establish normal ranges, as well as correlations with parameters associated with obesity/ insulin resistance.

All but one of the correlations we used were previously published. Altogether we finished with 10 correlations, as the various populations were subdivided according to sex, and in one study, menopausal status also.

All of the 10 correlations were *inconsistent* with a set‐point model of regulation. Like the population distributions of FT4/TSH the population distributions of calcium/PTH showed negative correlations between the levels of calcium and PTH, whereas a set‐point model implies positive correlation. The correlations between glucose and insulin were positive, but, as discussed above, in this system a set‐point model implies a negative correlation. Table 1 summarizes this information.

**Table 1:** Summary of empiric correlations derived from the literature and expected correlations in set‐point models of regulation. NS = not significant

Parameters	Population	*N*	Correlation	Correlation implied by set‐point model
Calcium/PTH Jorde et al. (1999)	Female	486	−0.195 (Pearson) (*P* < 0.01)	>0
	Male	460	−0.120 (*P* < 0.01)	>0
Calcium/PTH Minisola et al. (1993)	Female premenopause	35	−0.353 (*P* < 0.001)	>0
	Female postmenopause	35	−0.064 (*P* = NS)	>0
	Male	45	−0.661 (*P* < 0.001)	>0
Glucose/log insulin Gibson et al. (1975)	Female	148	0.37 (*P* < 0.001)	<0
	Male	142	0.06 (*P* = NS)	<0
Glucose/insulin Peplies et al. (2014)	Male	3640	0.39 (Pearson) (*P* < 0.001)	<0
	Female	3434	0.42 (Pearson) (*P* < 0.001)	<0
Glucose/log insulin Chen et al. (1999)	Male 1447, Female 1800	3247	0.0249 (Pearson) (*P* = 0.0001)	<0

## Discussion

We have extended our findings with regard to thyroid homeostasis to calcium and glucose homeostasis. We have shown that in both of these systems, all the population correlations are in the opposite direction to those of the relationship describing the effect of the controlling hormone on the parameter of interest. In all of these systems the population correlations are therefore *inconsistent* with a set‐point model of regulation as currently conceived. Our findings imply that the regulation in these systems is based on an equilibrium point rather than a set point. Alternatively, the concept of set points requires significant revision to accommodate the population data correlations. Our findings indicate that if an artifact or unidentified physiological relationship allowed a set‐point model to have a discrepant correlation in FT4/TSH homeostasis, this or another artifact or unidentified relationship must therefore also be present in Ca++/PTH and glucose/insulin homeostasis.

In the circumstances of homeostasis being based on an equilibrium point rather than a set point population, correlations may be in any direction, including the direction that is also consistent with there being a set point (Fitzgerald and Bean 2016). However, it is not merely a chance finding that none of our correlations were consistent with both models. Homeostasis is best served with there being restricted intraindividual variation in the physiology of the response of the controlling hormone to the parameter of interest, as this results in a corresponding restriction on the range of potential equilibrium values of that parameter of interest. Such restrictions lead to the empiric correlations (Fitzgerald and Bean 2016; Fitzgerald et al. 2017).

As the set‐point model of regulation implies strict requirements for the population distributions, this model implies that different subpopulations must have similar distributions in terms of the direction of the correlation. The finding of any population data not consistent with the set‐point model casts doubt on the validity of that model. The equilibrium‐point model of regulation however is consistent with any population distribution (Fitzgerald and Bean 2016), and with there being differences between the distributions of different subpopulations. Therefore our data, though limited, is sufficient to indicate that the regulation of serum calcium and glucose is consistent with an equilibrium‐point model but not with a set‐point model. Therefore, at a minimum, our work shows that not all homeostatic systems are based on set points.

Our conclusions depend on the integrity of the population data; that is, in a set‐point model, a normal individual's level reflects that individual's set point. In the absence of this assumption the whole concept of a set point becomes meaningless. The large samples of individuals supplying the calcium/PTH and glucose/insulin data were selected as representing the normal population and it would therefore seem unlikely that the data were corrupted by the presence of sufficient occult pathology to render the normal range parameter levels pathological rather than physiological. In any event any such pathology (e.g., complicating pathological insulin resistance) would need to be dependent on set points to be capable of distorting a correlation originally supportive of set point physiology. Furthermore, the largest study we located (Peplies et al. 2014) provided glucose/insulin data from a population of healthy children.

Systems in which there is prominent spiking of a parameter's level in response to a spike in the regulating hormone are by definition less stable and less suitable for this type of analysis. Female reproductive hormone physiology, testosterone/LH in men, and cortisol/ACTH physiology, for example, may require a different approach. In these systems there have been shown to be a lack of correlation (Rosenfield et al. 1977), or correlations consistent with either a set point or equilibrium‐point model (Bando et al. 1991; Foresta et al. 1997; Miro et al. 2004). However such systems are not regarded as homeostatic (Modell et al. 2015). In contrast the control of glucose and calcium are classical examples of homeostatic negative feedback (Modell et al. 2015), and therefore our findings suggest that homeostasis, in general, is based on equilibrium points rather than set points.

We know of no evidence indicating that the equilibrium‐point model is insufficient to explain all extant data, such that the proposal of a set‐point model is necessary. Our work adds to the work supporting an equilibrium model of homeostasis. Our conclusions do not affect the veracity of the many details of physiology previously documented. These are all equally valid in a set‐point, or an equilibrium point, model of regulation (Romanovsky 2005). It is perhaps for this reason that experimental data regarding physiological processes has previously only led to debate regarding the fundamental nature of set points/equilibrium points (Partridge 1982; Caputa 2005; Romanovsky 2005; Cabanac 2006). Conversely our results are not affected by the same details of physiology, for example, receptor physiology, hormonal metabolism etc (Fitzgerald and Bean 2016; Fitzgerald et al. 2017).

Our conclusions are consistent too, with evolutionary principles (Partridge 1982; Dumont et al. 2015). Though it is possible that an example of set point physiology might exist in the body we consider it to be more likely that all homeostatic processes evolved similarly (Partridge 1982). Our results are consistent with Claude Bernard's proposal regarding the stability of the milieu interieur; the principles of Walter Cannon, already modified (Goldstein 2009), however, would need further adjustment.

Though it is natural and intuitive to imagine that homeostasis works in a manner analogous to a thermostat or a cruise control system, objects we are familiar with, there may in fact be no everyday machinery that functions identically to a given physiological system (Partridge 1982; Modell et al. 2015). We suggest that, rather than extrapolating the characteristics of a real everyday system, to create an unreal analogy of homeostasis, it would be preferable to create a real analogy for homeostasis, by extrapolating the mechanism of homeostasis, to an everyday system.

We propose the following analogy/model as an example.

Rather than imagining the standard example of a set point driven heating system and a set point of 21°C, imagine a house heated by a large radiator with four elements. There is no thermostat to set in the house but one element of the radiator is programmed to come on when the temperature drops below 22°C. A second element becomes active when the temperature drops below 20°, a third below 18° and the fourth below 16°. Thus, at a temperature below 16° all four elements are active and the heater is working maximally.

Imagine further that we have a situation whereby the temperature of the house is stable at 21°. One element of the heater is active and heat losses equal heat gain. With a drop in the ambient external temperature heat losses increase and the temperature of the house drops to below 20°. At this point, the second element of the heater comes on. The house may then heat to above 20° and return to baseline heating; alternatively, depending on the heat loss properties of the house, the added heat may merely manage to keep the temperature steady at 19°, and thus a new point of stability is reached, with two elements of the heater active. There is not, in this latter situation, a sufficient stimulus to turn on another element which might restore the temperature to 21°. This response is not to be expected in the traditional thermostat controlled heating system whereby all the power necessary to restore the set temperature is deployed.

Alternatively again, depending on the size and insulation of the house, the extra heating may be insufficient to prevent the temperature of the house dropping further, to below 18 degrees. At this point, the third element of the heater is activated and again the resulting effect depends on the design of the house.

The system we propose responds to the temperature but it cannot alone determine the internal temperature which relies on the ambient external temperature, the heating system and other properties of the house. The temperature of the house is nevertheless protected to some extent by the heating system. By way of comparison the traditional thermostat model has only the options of the heater running with zero or four elements active,^1^ and the option of keeping the temperature steady at the set temperature of 21° (unless the system is overwhelmed). The internal temperature in the set point model is independent of the ambient external temperature and the properties of the house, the heating system compensating (again, unless overwhelmed) for any variations in heat loss properties.

Such a system as we have proposed may appear to behave similarly to a thermostat driven system even though there is no programmed temperature in the system. The responses of the radiator to changes in temperature are similar to those of a thermostat but the system as a whole does not “know” a desired or “goal” temperature. Instead each house's internal temperature results simply from the balance of the interplay of heat producing and heat loss mechanisms. If most houses in a neighborhood were reasonably similar, had similar radiators and the perturbations in heat loss were modest, the ambient temperature in all of the houses would be stable and similar, and this might create a resemblance of a “goal” temperature.

Further imagine two towns, both having a range of different types of house with different heat loss characteristics, but all the houses of one town are equipped with identical thermostatically (set point) controlled heating systems. These set points are set at different levels depending on the thermal comfort preferences of the inhabitants, and without regard to, (and so are independent of), the heat loss properties of the houses/the potential cost of heating etc. The other town has identical heating systems of the type described in our analogy (equilibrium). It now becomes possible to demonstrate the different relationships in the two towns between the internal temperature of the house, and heating energy consumed.

In the first town with a set point system, whereby the heating systems are driven to maintain a given temperature, as more energy is required to attain a higher temperature, on average the houses in which the set point is higher will have greater energy consumption. Thus, there will be a positive relationship between average temperature and energy consumption. This reflects our usual experience.

In the second town, however, the temperature of the houses is not governed entirely by the heating system, and those houses with greater heat losses will run at lower internal temperatures than those houses with smaller heat losses. These houses running lower temperatures will have the higher energy consumption, as the heating system reacts to, and compensates for, the lower temperatures by the mechanism of the radiator having more heating elements activated. Thus, there will be a negative relationship between the internal temperature and energy consumption.

We believe the type of heating system we propose in our analogy, (though nonexistent in practice to our knowledge), heuristically describes the homeostatic process and demonstrates the difference between physiology and our usual models of engineering. In a physiological context the components of the heating system would not be so discrete, such that there would be a continuous rather than a step‐wise response to temperature. There would therefore be no confusion with a model of multiple thermostats. Such a system applied to physiological systems is consistent with all of the observations recorded to date, including there being parameters controlled very tightly or not so tightly (Andersen et al. 2002), there being an impression of “heritability of set points” (Hansen et al. 2004) and there being alterations in “set points” with polymorphisms in components of the feedback loops (Medici et al. 2011).

Our work provides strong evidence that, an equilibrium‐point model of homeostasis is more consistent with empiric data than is a set point model. This proposition, previously proposed and supported by few authors (Partridge 1982; Romanovsky 2004), provides more of a physiological understanding than an impetus to major changes to clinical medicine. This clarification should, however, help reorientate research into, and the teaching of, basic endocrine physiology. Just as the removal of the Earth from the center of the solar system by Copernicus allowed for the removal of belief in the existence of crystal spheres, simple mechanical devices believed to hold stars and planets in their places (Romanovsky 2005), and just as the appreciation of the elliptical nature of planetary orbit paths allowed the disappearance of the concept of epicycles (Gearhart 1985), the removal of the set point from the center of the processes of regulation and homeostasis allows for the simplification of the understanding of these processes, and resolution of the problems referred to in the Introduction. There becomes no need to, identify or explain the set point and the controller involved in glucose homeostasis (Modell et al. 2015), seek the molecular and cellular mechanisms of set points in general (Modell et al. 2015), postulate hierarchies of control, differentiate regulated and controlled variables (Modell et al. 2015) or invoke allostasis (Goldstein, 2009) or other mechanisms to explain shifting set points (Modell et al. 2015). In systems that are not strictly homeostatic the simultaneous co‐existence of feedback and feed forward control becomes readily comprehensible. All parameter levels simply become the result of the various forces and feedback loops operating.

The further exposition of the above and the clinical implications of our analysis are the subjects of future planned work.

## Conflict of Interest

None declared.

## Note

1 This logic still applies in the circumstances of more modern set points, as seen in some modern heaters and cruise controls, that result in a proportionate response to an error signal, rather than the simple traditional on/off response.
